# Bridging from Intramuscular to Limb Perfusion Delivery of rAAV: Optimization in a Non-human Primate Study

**DOI:** 10.1016/j.omtm.2019.01.013

**Published:** 2019-02-02

**Authors:** Alisha M. Gruntman, Gwladys Gernoux, Qiushi Tang, Guo-Jie Ye, Dave R. Knop, Gensheng Wang, Janet Benson, Kristen E. Coleman, Allison M. Keeler, Christian Mueller, Louis G. Chicoine, Jeffrey D. Chulay, Terence R. Flotte

**Affiliations:** 1 University of Massachusetts Medical School, Worcester, MA 01655, USA; 2 Tufts University Cummings School of Veterinary Medicine, North Grafton, MA 01536, USA; 3 Applied Genetic Technologies Corp., Alachua, FL 32615, USA; 4 Lovelace Respiratory Research Institute, Albuquerque, NM 87106, USA; 5 Powell Gene Therapy Center Toxicology Core, University of Florida, Gainesville, FL 32610, USA; 6 Center for Gene Therapy, Nationwide Children’s Hospital, Columbus, OH 43205, USA

## Abstract

Phase 1 and phase 2 gene therapy trials using intramuscular (IM) administration of a recombinant adeno-associated virus serotype 1 (rAAV1) for replacement of serum alpha-1 antitrypsin (AAT) deficiency have shown long-term (5-year) stable transgene expression at approximately 2% to 3% of therapeutic levels, arguing for the long-term viability of this approach to gene replacement of secreted serum protein deficiencies. However, achieving these levels required 100 IM injections to deliver 135 mL of vector, and further dose escalation is limited by the scalability of direct IM injection. To further advance the dose escalation, we sought to bridge the rAAV-AAT clinical development program to regional limb perfusion, comparing two methods previously established for gene therapy, peripheral venous limb perfusion (VLP) and an intra-arterial push and dwell (IAPD) using rAAV1 and rAAV8 in a non-human primate (rhesus macaque) study. The rhesus AAT transgene was used with a c-myc tag to enable quantification of transgene expression. 5 cohorts of animals were treated with rAAV1-IM, rAAV1-VLP, rAAV1-IAPD, rAAV8-VLP, and rAAV8-IAPD (n = 2–3), with a dose of 6 × 10^12^ vg/kg. All methods were well tolerated clinically. Potency, as determined by serum levels of AAT, of rAAV1 by the VLP method was twice that observed with direct IM injection; 90 μg/mL with VLP versus 38 μg/mL with direct IM injection. There was an approximately 25-fold advantage in estimated vector genomes retained within the muscle tissue with VLP and a 5-fold improvement in the ratio of total vector genomes retained within muscle as compared with liver. The other methods were intermediate in the potency and retention of vector genomes. Examination of muscle enzyme (CK) levels indicated rAAV1-VLP to be equally safe as compared with IM injection, while the IAPD method showed significant CK elevation. Overall, rAAV1-VLP demonstrates higher potency per vector genome injected and a greater total vector retention within the muscle, as compared to IM injection, while enabling a much greater total dose to be delivered, with equivalent safety. These data provide the basis for continuation of the dose escalation of the rAAV1-AAT program in patients and bode well for rAAV-VLP as a platform for replacement of secreted proteins.

## Introduction

Alpha-1 antitrypsin (AAT, or SERPINA1) is a highly abundant, 52-kDa serum protein. It is a multifunctional antiprotease, a member of the serpin gene superfamily, which functions primarily to inhibit the activity of neutrophil elastase (NE) and other neutrophil proteases.^1^, ^2^ Deficiency of AAT leads to progressive lung disease, due primarily to the unopposed action of NE and other neutrophil products on the extracellular matrix in the pulmonary interstitium. NE degradation products are also pro-inflammatory, leading to enhanced recruitment of neutrophils to the lung in response to infection or other inflammatory stimuli, such as cigarette smoke. Thus, patients with AAT deficiency suffer from progressive chronic obstructive pulmonary disease with panacinar emphysema as the elastic recoil of the lung is lost. There is a strong founder effect among Europeans with AAT deficiency, with the E342K (PiZ) missense mutation accounting for over 90% of disease-causing alleles. The allele frequency of PiZ is 1% to 2% in the European population.^3^ Current therapy consists largely of protein replacement (augmentation therapy) and is available in the United States and some European countries, with the approved dosing interval currently weekly by intravenous infusion, leading to a substantial desire among patients for gene therapy to be developed. Normal adult serum AAT levels range from 20 to 53 μM,^2^ and the threshold to prevent lung disease is 11 μM, making it the most abundant serum protein for which gene therapy has been attempted.

This work is a continuation of pre-clinical and clinical work to develop a gene therapy for AAT deficiency. This work has focused on a muscle-based gene therapy platform using a recombinant adeno-associated virus serotype 1 (rAAV1)-CB (chicken beta-actin promoter with a CMV enhancer)-human alpha-one antitrypsin (hAAT) vector to enable systemic secretion of the normal (wild-type human alpha-one antitrypsin [PiM], M-AAT) version of the AAT protein in AAT-deficient patients. The endpoint for licensure of rAAV1-CB-hAAT by the Food and Drug Administration (FDA) is based on the serum level of AAT that has been shown to be protective from lung disease. This threshold is currently set at 11 μM of M-AAT in the patient serum or plasma. The delivery of rAAV1-CB-hAAT to the muscle of AAT-deficient patients in previous trials by our group has proven to be safe and has demonstrated a dose-response relationship with a maximum patient serum level of approximately 300 nM. The AAT serum levels have proven stable for 5 years after a single set of 100 intramuscular (IM) injections with a vector dose of 6 × 10^12^ vg/kg in a volume of 135 mL.^4^, ^5^, ^6^ Our previous patient dose-escalation results suggest that further increases in vector dose would result in elevations in serum M-AAT levels in an approximately linear fashion. However, increases in dose are limited by the fact that the vector formulation cannot be concentrated significantly further and that an increase in the number of direct IM injections is not tolerable to patients.

Based on these considerations, we have designed this as a bridging study in rhesus macaques to compare direct IM injections to local intravascular infusion. For this work, we utilized a c-myc-tagged rhesus AAT gene so that the relative expression levels could be quantitatively compared. We chose to compare two intra-vascular delivery protocols that have been previously published as efficacious routes of AAV administration to large animal models (Table S1). The first method involves intra-arterial delivery with a double balloon catheter to the pelvic extremity, a method that has been successfully used in non-human primates.^7^ Variations of the intra-arterial technique have also been successfully used for AAV delivery in both non-human primates and canine models (Table S1).^8^, ^9^, ^10^, ^11^, ^12^, ^13^ We compared the intra-arterial delivery to high-volume venous limb perfusion (VLP; also called hydrodynamic intravenous delivery, or HID) into a tourniqueted pelvic extremity.^14^, ^15^, ^16^, ^17^, ^18^, ^19^, ^20^, ^21^, ^22^, ^23^ VLP has also been successfully used for AAV delivery in both canids and non-human primates (Table S1). The intra-arterial push and dwell (IAPD) and intravenous (VLP) delivery methods were compared to direct IM injection using an AAV rhesus AAT-c-myc vector (n = 3 per group for the rAAV1 IM group, rAAV1 VLP group, and rAAV8 VLP group; n = 2 for the rAAV1 IAPD and rAAV8 IAPD groups) (Table 1). All animals were given the same dose of AAV. The IAPD vector was given in a total vector volume of 12 mL/kg, while the VLP vector was given in a total volume of 50 mL/kg. We chose the AAV serotypes for this study to bridge our current clinical program, in the case of AAV1, and because of its utilization for both intravenous and intraarterial limb perfusion previously in the case of AAV8 (Table S1). Only AAV1 was given IM to directly compare the current AAV1 IM clinical program to AAV1 delivered by limb perfusion.

**Table 1:** Rhesus Limb Infusion Experimental Design

Dose Group	Animal # (Sex; NAb titer)	Test Article (6 × 10^12^ vg/kg for All Groups)	Route	Dosing Volume
IM-AAV1	RA1683 (F; 1:5)	rAAV1-CB-rhAATmyc	IM	0.5 mL per injection (8 injections)
	RA1598 (M; 1:10)			
IAPD-AAV1	RA1709 (F; 1:5)	rAAV1-CB-rhAATmyc	IAPD	12.5 mL/kg
	RA1562 (M; 1:10)			
IAPD-AAV8	RA1660 (F; 1:10)	rAAV8-CB-rhAATmyc	IAPD	12.5 mL/kg
	RA1664 (F; 1:10)			
VLP-AAV1	RA0770 (F; <1:5)	rAAV1-CB-rhAATmyc	VLP	50 mL/kg
	RA1567 (M; 1:10)			
VLP-AAV8	RA1676 (F; 1:10)	rAAV8-CB-rhAATmyc	VLP	50 mL/kg
	RA1703 (F; 1:10)			

All dosing was performed in the right hindlimb. Intramuscular dosing was performed as a total of eight injections, four into the right quadriceps and four into the right gastrocnemius. NAb, neutralizing antibody titer (anti-AAV1 or anti-AAV8 titer listed depending on the vector delivered to that animal); IM, intramuscular; F, female; M, male; CB, chicken beta-actin promoter with a CMV enhancer; rhAATmyc, rhesus alpha-1 antitrypsin gene with a c-myc tag; IAPD, intra-arterial push and dwell; VLP, venous limb perfusion;

VLP resulted in higher serum AAT levels when compared to IM (AAV1)- and IAPD (AAV1 and AAV8)-dosed animals. IAPD using AAV1 had comparable serum levels to those of animals dosed by IM injection at day 60. These data support the use of VLP as a viable delivery route for AAT gene therapy to skeletal muscle, allowing comparable to improved expression and increased dosing scalability due to the increase in the deliverable vector volume.

## Results

### Limb Infusion Procedures

**Figure 1:**
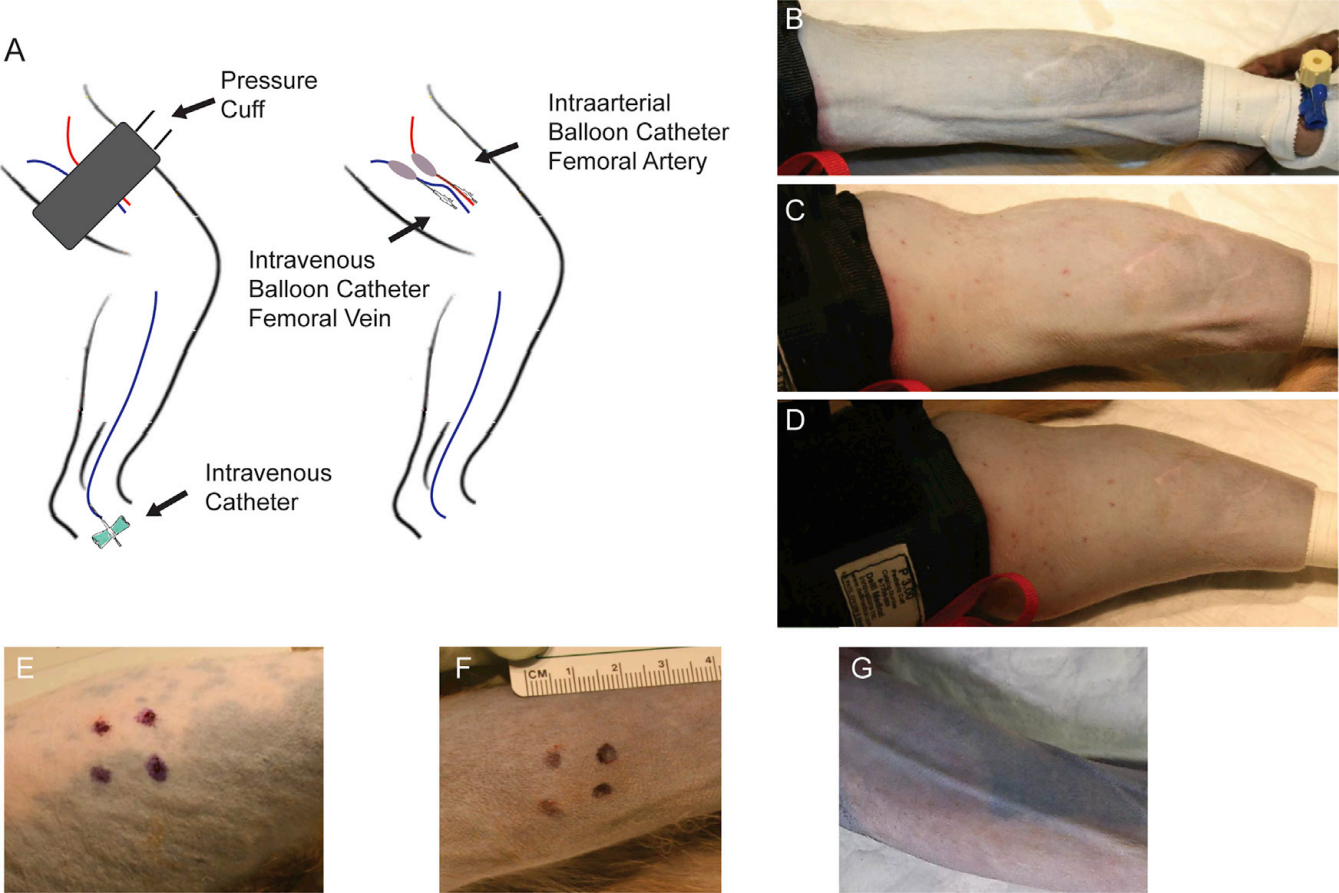
Representation of Limb Perfusion Routes and Images of the Dosed Limbs for Each Route of Delivery (A) Graphical representation of venous limb perfusion (left) and intra-arterial push and dwell (right). (B–D) VLP dosed limb (B) at the start of delivery, (C) 5 min into the vector infusion, and (D) after all vector had been infused but tourniquet had not been released. (E) Quadriceps dosing sight; note that intra-muscular (IM) injection sights are denoted using a black marking pen. The animal’s body is to the left of the image and the knee is to the right. (F) Gastrocnemius dosing sight; note that IM injection sights are denoted using a black marking pen. The animal’s knee is to the left of the image, and the foot is to the right. (G) Intra-arterial push and dwell (IAPD)-dosed limb at the end of infusion before balloon catheters deflated (the image shows the inner aspect of the dosed limb).

The limb perfusion methods used for this study are diagrammed in Figure 1A. All animals tolerated both procedures well and recovered without incidence. The IAPD procedure had a total procedure time of around 4 h and required three surgical personnel, one anesthetist, and two technical assistants to perform. The VLP procedure had a total procedure time of around 1 h and required two technical assistants and one anesthetist to perform. The increased procedural time with the IAPD procedure resulted from the time to place the catheters surgically and the time to confirm catheter placement by fluoroscopy. Marked limb swelling was seen following the VLP procedure, but this resolved completely within 12–24 h post-procedure and did not alter the animal’s ability to use the limb normally, even immediately post-anesthesia (Figures 1B–1G).

### Quantification of rhAAT-c-myc by Semi-Quantitative Immunoblot of Serum

**Figure 2:**
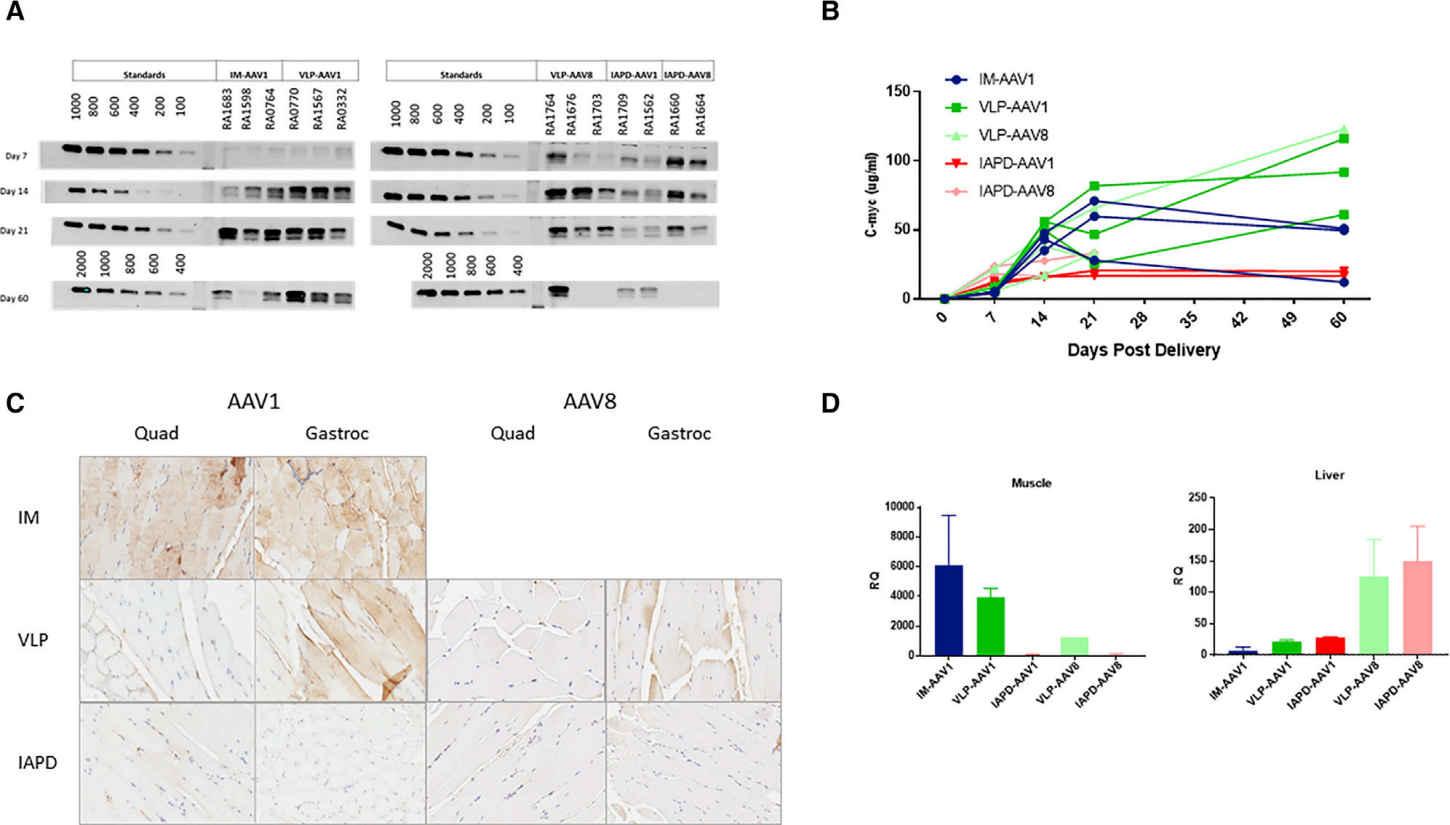
Serum AAT c-myc Expression, Muscle Immunohistochemistry for AAT, and RT-PCR (A) Western blot gel image for all time points (please note that several AAV8 day-60 samples were missing). (B) Semi-quantitation of western blot (WB) data represented as mean ± SEM. (C) Immunohistochemistry for AAT at day-60 representative sections for each dosing route for both AAV1 and AAV8. Muscle samples were collected for immunohistochemistry (IHC) from the level of the IM injections in both the quadriceps and gastrocnemius. (D) RT-PCR data from day-60 muscle and liver tissue. The gastrocnemius muscle samples were used for all groups. IM muscle samples were collected from the injection site. IM, intramuscular; IAPD, intra-arterial push and dwell; VLP, venous limb perfusion.

Taking advantage of the c-myc epitope tag, we developed a semiquantitative serum immunoblot method for the detection of the rhesus AAT (rhAAT)-c-myc fusion protein, titrated against a series of dilutions of a known standard. Immunoblots were performed using serum collected at days 7, 14, 21, and 60 post-vector delivery (Figure 2A). Semiquantitative levels at each time point were then determined by chemi-luminescence, and the value plotted in Figure 2B. VLP delivery of either rAAV1 or rAAV8 yielded an approximately 2-fold increase in total serum transgene expression at day 60 when compared to day-60 rAAV1 IM-injected animals (Figures 2A and 2B). Serum levels following IM injection of AAV1 were comparable to those for animals dosed with AAV1 via the IAPD route at day 60. It should be noted that some animals in the AAV1 IM groups were noted to have decreased serum levels of rhAAT-c-myc at day 60 compared to day 21. None of the differences were statistically significant.

### Muscle Immunohistochemistry for AAT

AAT muscle expression was greatest around the injection sites for the IM dose group. We saw widespread low-level expression in the IAPD group for both the quadriceps and gastrocnemius (Figure 2C). We observed increased expression in the gastrocnemius region for the VLP group, with lower levels of positive myocytes in the quadriceps (Figure 2C).

### Real-Time qRT-PCR

Primers targeting the AAT-c-myc junction were utilized to identify transgene RNA expression in the gastrocnemius muscle (from the site of injection in the IM-dosed animals) and liver at day 60 post-delivery. Muscle expression was higher in the IM and VLP groups compared to the IAPD groups (Figure 2D). In the liver, RNA levels were highest in the VLP-AAV8 and IAPD-AAV8 groups (Figure 2D). All AAV1 dosing groups had similar liver expression. Muscle expression was markedly higher than liver expression in all the AAV1 dosing groups.

### Total Vector Genomes

**Figure 3:**
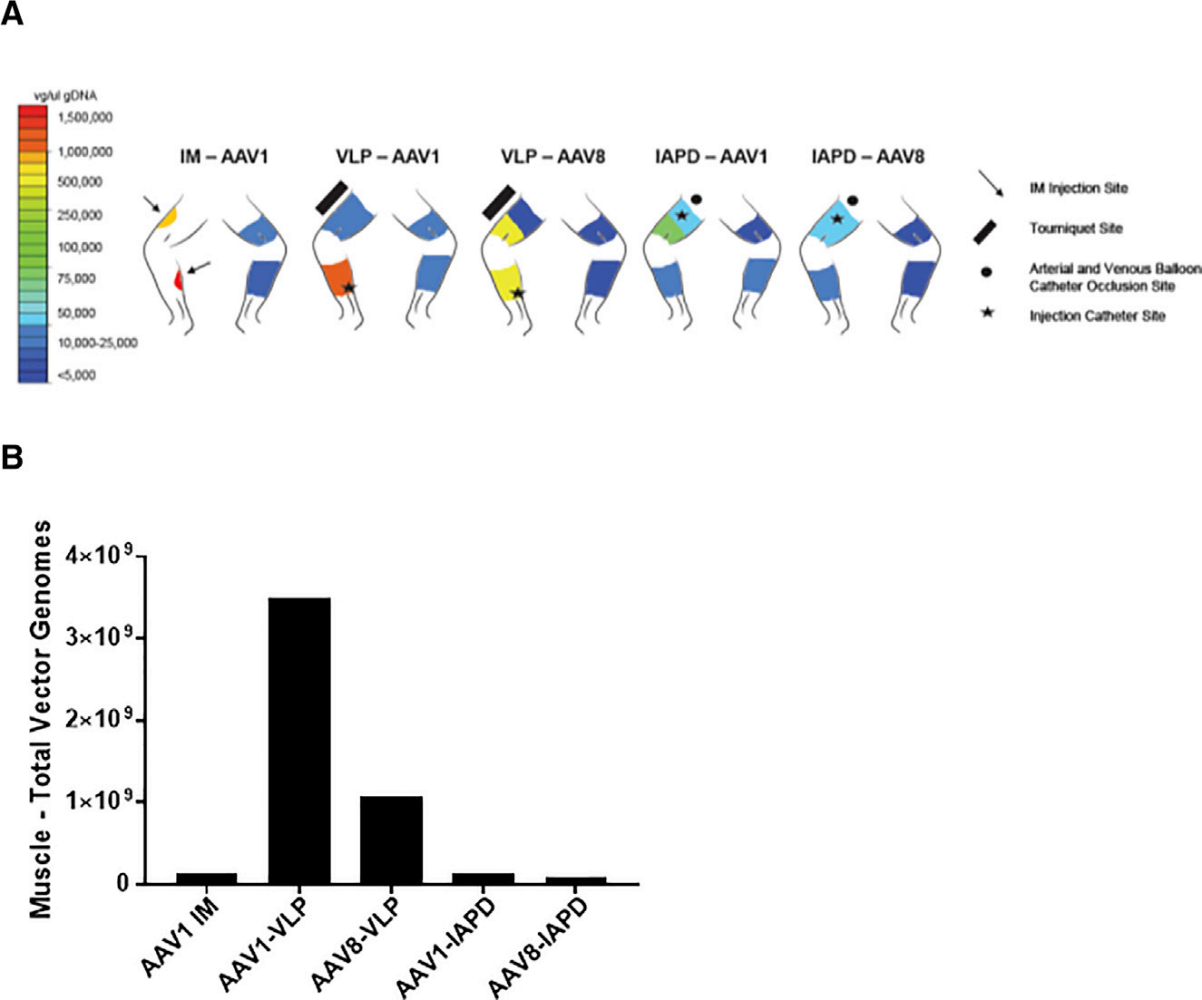
Vector Distribution in the Muscle and Estimated Total Vector Genomes in the Lower Extremity (A) Heatmap of distribution of vector genomes in the muscle of both the dosed and undosed limbs 60 days post-delivery. IM, intramuscular; IAPD, intra-arterial push and dwell; VLP, venous limb perfusion. n = 2 per group. (B) Total vector genomes delivered to the targeted rhesus lower extremity muscle, as estimated based on the volume of muscle tissue perfused and the nuclear density of muscle tissue.

In order to confirm that the increased expression observed in the rAAV1-VLP group was due to an increase in the total number of vector genomes delivered to the lower extremity musculature, we performed qPCR for rAAV vector genomes, normalized to the quantity of genomic DNA (i.e., micrograms of gDNA) (Figure 3A; Table 2). The volume of muscle transduced was estimated using the estimated volume of the limb perfused by the vessel cannulated. In the case of IM, the volume of injection was used as an estimate of the volume of muscle tissue transduced, based on prior studies including real-time ultrasound performed during deltoid muscle injections in humans in a previous trial performed by our group.^24^ The number of myofiber nuclei constituting that volume was then estimated using the estimate of nuclear density previously published.^25^ This comparison suggested a superiority of rAAV1-VLP delivery over rAAV1-IM and other modes of muscle delivery. In fact, the number of vector genome (vg) copies delivered to the muscle was estimated at 25 times greater with rAAV1-VLP than with rAAV1-IM (Table S2).

**Table 2:** Tissue Vector Genomes

Tissues (Vector Genomes/μg gDNA)								
**Mean (SD)**								

**Serotype-Route**	**Right Upper Quadriceps (Dosed Limb)**	**Right Lower Quadriceps (Dosed Limb)**	**Right Calf (Dosed Limb)**	**Right Inguinal Lymph Node (Dosed Limb)**	**Liver**	**Left Quadriceps (Undosed Limb)**	**Left Calf (Undosed Limb)**	**Left Inguinal Lymph Node (Undosed Limb)**

AAV1-IM	498,778 (281,500)	574,208 (562,329)	1,599,935 (1,115,968)	2,532,248 (1,319,764)	376,475 (387,624)	9,878 (11,388)	993 (1,118)	37,998 (30,675)
AAV1-IAPD	44,150 (37,218)	129,055 (127,706)	22,530 (18,723)	368,133 (111,128)	1,192,300 (284,074)	3,008 (2,120)	9,150 (5,185)	35,348 (25,648)
AAV8-IAPD	86,205 (91,689)	70,038 (25,065)	16,885 (8,792)	293,298 (103,261)	2,878,100 (1,665,077)	3,028 (2,099)	4,253 (2,648)	55,638 (47,087)
AAV1-VLP	10,188 (8,071)	9,045 (5,069)	783,635 (87,715)	2,213,788 (90,284)	1,961,980 (470,336)	13,040 (11,487)	9,025 (6,956)	92,483 (39,652)
AAV8-VLP	1,775 (526)	203,595 (121,972)	239,328 (84,476)	980,443 (19,295)	6,534,063 (5,428,745)	2,070 (319)	5,353 (4,253)	131,755 (–)

IM, intramuscular; IAPD, intra-arterial push and dwell; VLP, venous limb perfusion.

Next, we compared the total number of vgs retained within the muscle (as described earlier) with the total number of vgs detected in the liver, assuming that the liver of a rhesus macaque contains approximately 4.5 × 10^10^ nuclei, a value based on data from Marcos and Correia-Gomes on the number of nuclei (from all cell types) per gram of mammalian liver (4.5 × 10^8^ nuclei per gram) and from Kim et al., indicating the average liver weight in a rhesus of approximately 100 g.^26^, ^27^ These data were then used to calculate the ratio (as a percentage) of the total vgs detected within the muscle, as compared with the total vgs detected within the liver, expressed as a percentage: (muscle vg/liver vg) × 100 (Table S2).

Interestingly, with direct IM injection of rAAV1-AAT, the total number of vg detected in the liver as a whole was estimated at 6.0 × 10^10^ vg, which is substantially greater than the amount retained within the muscle, which was 1.37 × 10^8^ vg. While rAAV1-VLP did result in a 3-fold increase in total vg within the liver (up to 1.9 × 10^11^ vg), the proportional estimated increase retained in the muscle was much greater at over 25-fold (3.5 × 10^9^ vg as compared with 1.37 × 10^8^ vg). Estimating the ratio of total vg in muscle as compared with liver, rAAV1-IM showed only 0.22% of the abundance in liver, while rAAV1-VLP showed muscle vg at 1.09% of the total detected in liver. This represents an estimated 5-fold increase in relative vg retention in muscle as compared with liver.

### Serum Chemistry and Complete Blood Count

**Figure 4:**
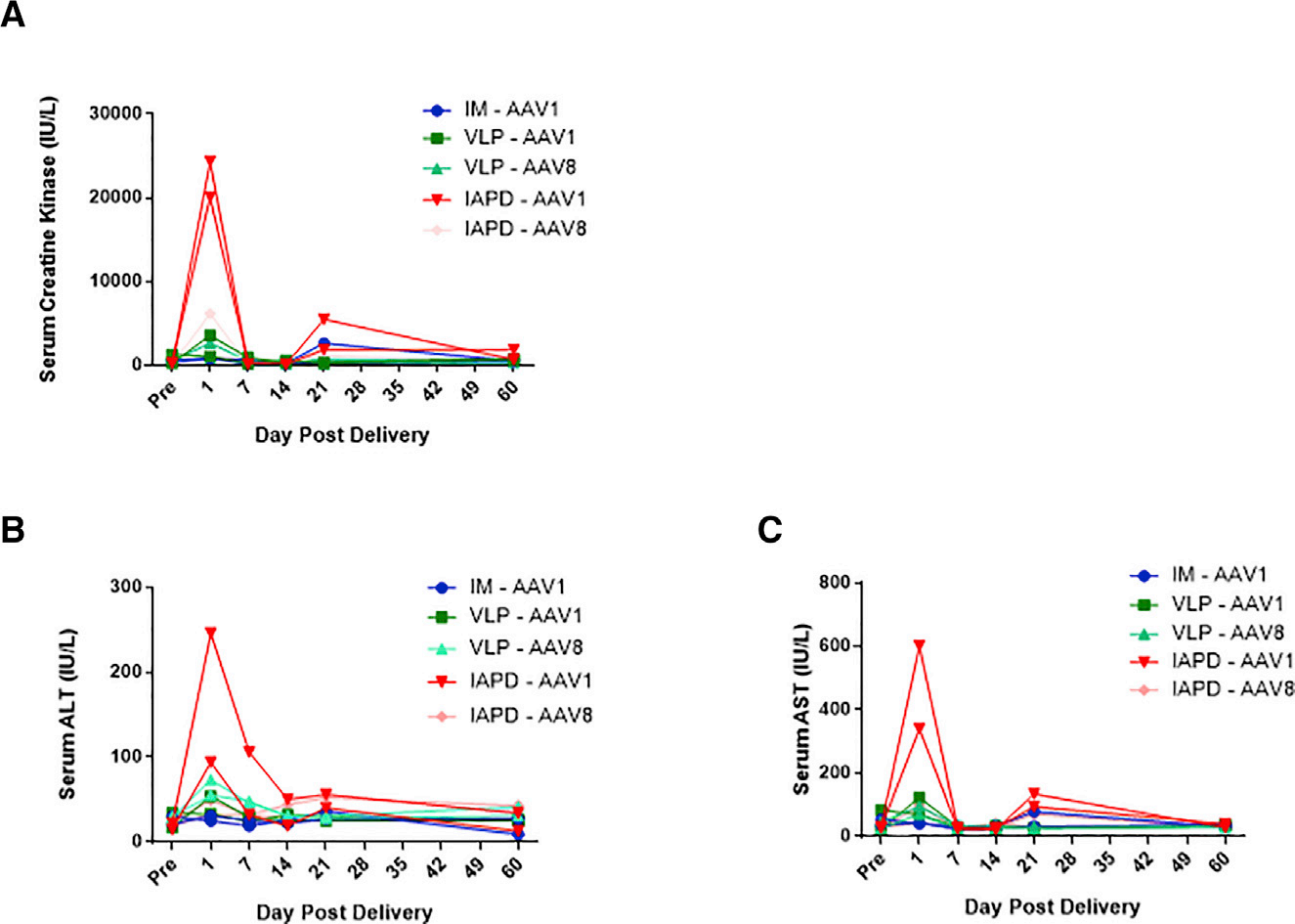
Serum Creatine Kinase and Liver Transaminase Levels following rAAV Rhesus AAT-c-myc Delivery (A) Serum levels of creatine kinase (CK), a marker of myocyte damage. (B) Serum levels of alanine aminotransferase, a transaminase that increases in the serum following hepatocellular damage. (C) Serum levels of aspartate aminotransferase, a transaminase that increases in the serum following hepatocellular damage. IM, intramuscular; IAPD, intra-arterial push and dwell; VLP, venous limb perfusion; ALT, alanine aminotransferase; AST, aspartate aminotransferase; AAV1 and AAV8, AAV capsid types delivered. n = 2 per group.

We saw a moderate spike in serum creatine kinase, a marker of muscle damage, 1 day after delivery and a mild spike 21 days after AAV1 IAPD delivery (Figure 4A). At day 1, all other groups had minimal to no increase in serum creatine kinase. The IM group had a very mild increase in CK in one animal at day 21. There was a moderate increase in serum alanine aminotransferase (ALT) and aspartate aminotransferase (AST) at day 1 post-delivery in the IAPD AAV1 group as well as a mild AST elevation at day 21 in that same group (Figures 4B and 4C). There were no other serum chemistry parameters or complete blood counts that changed significantly following vector dosing.

### IFNγ-ELISpot Response to AAV1 and AAV8 Capsids

The T cell response to both AAV1 and AAV8 capsids were monitored by IFNγ ELISpot assay (Table S3). Peripheral blood mononuclear cells (PBMCs) were expanded for 6 days prior to the assay. Our results showed that none of the animals injected IM had a positive response prior to dosing and at day 60 post-delivery. One out of 3 animals showed a mild positive response to the AAV1 capsid at day 60 post VLP dosing (less than 350 spot-forming units [SFUs] per million of cells) (Figure S1; Table S3). Regarding the animals injected with the AAV8 vector, none of the animals injected via VLP had an IFNγ-positive response to the capsid prior to or post-dosing. One animal injected via AAV8 IAPD showed a mild positive response at necropsy (less than 200 SFUs per million of cells) (Figure S2; Table S3). Our results suggest there is no or very limited systematic cellular immune response to either AAV1 or AAV8 capsids after IM, VLP, and IAPD vector administration.

## Discussion

The present study provides the first direct comparison in non-human primates of several previously published methods of delivery of rAAV vector to limb musculature and effectively bridges the rAAV1-AAT muscle delivery program from direct IM to VLP. All methods were safe in terms of the overall clinical well-being of the animals. Expression levels similar to or higher than those for IM injection were obtained using each of the limb perfusion methods. The rAAV1-VLP method was the most efficient, with a 25-fold increase in total vg delivered to the muscle tissue and double the total vector transgene expression. The rAAV1-VLP method did not result in a significant increase in muscle injury compared with rAAV1-IM, as measured by serum CK levels and histology. The VLP method was also safer than rAAV1-IAPD, which did demonstrate a short-term spike in serum CK values 1 day after vector injection. Liver transaminase elevations were also noted in the rAAV1-IAPD group but not in the other dosing groups. The rAAV1-VLP also preserved the preference of delivery for muscle over liver, as demonstrated by the fact that the total vector DNA amount detected in the muscle was much greater in the rAAV1-VLP group as compared with the rAAV1-IM group (25-fold), while the increase in liver vector DNA with VLP was only 3-fold. Meanwhile, the same VLP method with rAAV8 resulted in a greater spread to liver than the rAAV1-VLP group but did not mediate higher expression. This suggests that the superiority of the rAAV1-VLP for overall serum AAT expression is derived from muscle rather than liver AAT expression. If the gene expression advantage had been due to liver delivery, then the rAAV8-VLP group would have been expected to express at a significantly higher level.

Interestingly, while direct IM injection has been assumed to result in muscle-restricted vector delivery, the proportion of total vector DNA retained in muscle as compared with that in liver in the rAAV1-IM group was only 0.22%. The same comparison with rAAV1-VLP showed that total vg retention in muscle was 1.09% of that observed in liver, about a 5-fold improvement in relative proportion. This is consistent with the observation of greater total serum AAT expression from the rAAV1-VLP group.

The safety profile of rAAV1-VLP was excellent. The approach did not result in significant elevations in muscle or liver enzymes or in an IFNγ T cell response to the capsid. A positive IFNγ T cell response was observed in only 2 out of 13 animals. It is noteworthy that these infrequent responses were mild and observed only after a cell expansion of 6 days, and they were directed to both serotypes 1 and 8 after VLP or IAPD administration, suggesting that the cellular immune response is not systematic and not related to a given serotype or method of delivery. Based on the results presented, we cannot rule out a transgene immune response; this will be assessed, along with cellular infiltrated into dosed muscle, in future experiments.

Overall, the rAAV1-VLP method appeared to be superior to rAAV1-IM and other limb perfusion methods in the overall effectiveness of transgene expression and safety at matched doses of vg per kilogram. We anticipate that a transition of the rAAV1-hAAT muscle-targeted clinical gene therapy program from IM to VLP will allow for an increased volume of vector to be delivered safely and effectively. Since vector concentration has already been previously maximized, the increase in volume allows a greater total dose of vector to be delivered to the human subjects’ muscle while avoiding the increased risks of arterial cannulation and the morbidity associated with a much large number of IM injections. This bridging from IM to VLP will effectively extend the work with the rAAV1-hAAT vector dose escalation without any significant increase in the risk to subjects from the administration.

## Materials and Methods

### Study Outline

Animals were selected based on serum neutralizing antibodies against AAV1 or AAV8 as determined commercially by the University of Massachusetts Gene Therapy Vector Core using an *in vitro* assay; animals with a titer at or below 1:10 were selected. Five groups of animals were administered rAAV1-CB-rhAATmyc or rAAV8-CB-rhAATmyc by IM, IAPD, or VLP as assigned (Table 1) on study day 0. The vector dose was 6 × 10^12^ vg/kg for all groups. After dosing, animals were monitored by veterinary staff twice daily for 7 days for pain, bleeding, suture loss, limping, or other signs. Detailed clinical observations and body weight were recorded. At study day 60, animals were euthanized and subject to a complete necropsy, and blood and tissues were collected for evaluation. All animal work was done under the approval and supervision of the Lovelace Respiratory Research Institute Animal Care and Use Committee.

### Vector Design and Manufacturing

The vector was manufactured by Applied Genetic Technologies Corp (AGTC) as previously described.^4^ Briefly, the vector used for these studies is an AAV2-inverted terminal repeat (AAV2-ITR) cassette, cross-packaged into AAV1 capsids. The enhancer-promoter complex is a commonly used constitutively active hybrid consisting of the CMV-immediate early enhancer, the chicken beta-actin promoter, and an intron from rabbit beta globin (CBA, alternatively known as CAG). The transgene used for these studies is the rhAAT with an in-frame fusion to the human-derived c-myc peptide fragment. Our lab has used c-myc fusions of the AAT gene derived from the native species for several previous preclinical studies including in baboons.^28^ The design enables one to quantify the transgene product expression levels while minimizing immunologic responses against the AAT gene.

The vector was packaged into AAV1 capsids using the recombinant herpes simplex virus 1 (rHSV1) -system.^29^, ^30^, ^31^, ^32^, ^33^ In this system, two separate rHSV1 stocks are used to infect baby hamster kidney (BHK) cells used for upstream production. One was rHSV1-Rep2Cap1. The other contained the AAV-CB-rhAATmyc expression cassette inserted into an ICP27 deleted HSV-1 virus (d27.1). Cell lysis with Triton X-100 detergent was followed by Benzonase treatment to remove unpackaged DNA. The treated lysate was filter clarified, concentrated, buffer exchanged, and then column purified using CIM Q Monolith anion-exchange chromatography followed by AVB Sepharose affinity chromatography. The final product was concentrated by tangential flow, exchanged to Ringer’s buffer, and passed through a 0.2-μm filter.

### Vector Dosing

#### Test Article Preparation

The vector was administered in volumes dictated by the injection or infusion procedure (Table 1). For each administration route, individual stock vials of vector were thawed and diluted on the day of use in the appropriate concentration and volume to deliver the targeted vector dose (6 × 10^12^ vg/kg). The vector was diluted with Lactated Ringer’s Solution.

### Intramuscular Injection

The animals were anesthetized with ketamine (10 mg/kg with 2–3 mg/kg bumps as needed) administered via IM injections. For the IM dose group, rAAV1-CB-rhAATmyc vector was administered as eight 0.5-mL injections (i.e., 4 mL of total dose volume), with the concentration adjusted to achieve the desired total dose based on the body weight of an animal. The injections were performed into the quadriceps and gastrocnemius muscles in the right hindlimb, with 4 injections in each muscle. The spacing between injections depended on the size of the muscle but were 0.5 to 1 cm apart. The injection sights were marked with a black marking pen for photography of the injected limbs. Post-injection pain, if observed, was managed with buprenorphine (0.01–0.03 mg/kg) administered via IM injection. Thereafter, buprenorphine (0.01 to 0.03 mg/kg, IM injection) was administered as needed, based on clinical observations.

### Intravascular Limb Infusion

#### Pre-surgical Preparation and Anesthesia

Aseptic technique was used throughout the surgical procedure for the IAPD and VLP delivery. For surgical procedures, animals were pre-medicated with ketamine (10 mg/kg) administered intramuscularly. Inhalant anesthesia (generally 1–4% isoflurane in oxygen for induction and 0.5–3% isoflurane in oxygen as needed for maintenance) was administered via face mask to facilitate intubation. During the operative procedure, anesthesia was maintained with 0.5–3% isoflurane in oxygen administered via the endotracheal tube.

One or two venous catheters were placed in a peripheral vein in the leg or arm (not the leg for the infusion). The catheters were used as needed to inject heparin and protamine, to withdraw blood for assessment of clotting time, and to provide Plasmalyte (5 mL/kg/h) during the infusion procedure.

#### Intra-Arterial Push and Dwell

IAPD animals received the vector (rAAV1-CB-rhAATmyc or rAAV8-CB-rhAATmyc) in a volume of 12.5 mL/kg of Lactated Ringer’s Solution.

Buprenorphine (0.01 to 0.03 mg/kg, administered via IM injection) was given preemptively at least 20 min prior to incising skin. The surgical site was prepared according to standard sterile procedure. After lidocaine (1 mg/kg) and bupivacaine (1 mg/kg) were administered by local application at the incision site, an incision was made in the lower anterior thigh of the right pelvic limb, and the superficial femoral artery and vein were dissected and isolated with silk suture. Arterial and venous access was obtained with sheath catheters. The catheters were inserted by the cut-down technique and then retrograde positioned into the upper femoral artery and vein near the inguinal ligament. The arterial and venous balloon catheters were then placed through their respective sheathes. The stopcock on the venous catheter was turned to prevent venous outflow. Correct placement of the catheters was checked by fluoroscopy, confirming the presence of the arterial and venous balloons above the level of the vascular branches leading to the quadriceps muscle groups. Once the vein and artery were cannulated, heparin was administered to achieve an activated clotting time of >350 s determined using an i-STAT clinical analyzer and an activated clotting time (ACT) cartridge. The limb was elevated and wrapped tightly to massage all venous blood from the limb, after which the catheter balloons were inflated to prevent the vascular flow of the femoral vein and artery. The limb was then lowered and unwrapped. After a pre-flush with Lactated Ringer’s Solution (LRS) (5 mL/kg), the vector (rAAV1-CB-rhAATmyc or rAAV8-CB-rhAATmyc) in a volume of 12.5 mL/kg was infused as quickly as possible though the arterial catheter sheath port. The vector solution dwelled for 15 min, after which repeat fluoroscopy confirmed that the balloons had remained inflated through the entire dwell time. At that point, a post-flush of 5mL/kg of LRS was injected into the arterial catheter. After the infusion, the balloons were deflated, and the catheters were removed. The effects of the circulating heparin were reversed by the injection of protamine (0.5–1 mg/100 United States Pharmacopeia [USP] heparin units administered). Blood samples were obtained, and clotting times were checked. When the clotting time had returned to near baseline value (±20 s), the animal was recovered from anesthesia and returned to its home cage.

#### VLP

VLP-dosed animals received the vector (rAAV1-CB-rhAATmyc or rAAV8-CB-rhAATmyc for group 5) in a volume of 50 mL/kg LRS.

For the VLP procedure, an intravenous catheter was placed into the distal peripheral saphenous vein of the right pelvic limb. The limb was elevated and wrapped tightly from distal to proximal (from just above catheter to mid-thigh) to massage as much blood as possible from the limb. A tourniquet was then placed around the level of the proximal thigh and tightened to prevent vascular flow into and out of the limb. The tourniquet extended from proximal to mid-thigh. The limb was then lowered and unwrapped. The vector (rAAV1-CB-rhAATmyc or rAAV8-CB-rhAATmyc) in a volume of 50 mL/kg was infused over about 5–10 min. The tourniquet remained tight for 15 min following the infusion and was then released. The catheter was removed, and the animal was allowed to recover from anesthesia and returned to its home cage.

#### Physiological Parameter Monitoring During Infusions

Heart rate, respiratory rate, and body temperature were monitored and documented during the surgical procedure to evaluate the status of animals.

#### Post-Vector Administration Monitoring and Observations

After vector administration on the dosing day, animals subjected to infusion procedures (groups 2–5) were observed for evidence of erythema and edema of the infused site; blood vessel rupture; compartment syndrome; traumatic rhabdomyolysis; high intravascular pressure; bleeding (hematoma); pain; abnormal gait limping; and potential damage to nerves, muscles, or the vascular network.

In addition, after vector administration, all animals (groups 1–5) were monitored for clinical signs twice daily for 7 days. Behavioral and clinical observations were made on awake animals, with special attention paid to the legs and any abnormal motor movements (including posture or gait abnormalities).

For serum chemistry analyses, blood was collected into a serum separator or clot tubes for centrifugation to separate cellular and serum fractions. Serum chemistry was determined using a Hitachi Modular Analytics Clinical Chemistry System (Roche Diagnostics, Indianapolis, IN, USA).

#### Quantification of rhAAT-c-myc by Semi-Quantitative Immunoblot of Serum

Serum samples and standard were diluted in 1:50 PBS. We mixed 10 μL diluted serum with 10 μL Tris-glycine SDS sample buffer (2× Novex) and heated at 85°C for 10 min. 20 μL treated sample was run on Novex 12% Tris-glycine gels (Invitrogen, Carlsbad, CA, USA) using Tris-glycine SDS running buffer (Invitrogen, Carlsbad, CA, USA). The protein was then transferred to nitrocellulose membranes using an i-Blot transfer device (Invitrogen, Carlsbad, CA, USA). Membranes were blocked for 1 h at room temperature with Odyssey Blocking Buffer (LI-COR Biosciences, Lincoln, NE, USA) before being probed overnight with primary antibodies (1:1,000 dilution) (goat c-myc antibody; GeneTex catalog no. 30518). Infrared dye (IR)-labeled secondary antibodies (1:5,000 dilution) were then applied using IRDye 680LT Donkey Anti-Goat IgG (H + L). Blots were visualized using the Odyssey Infrared imaging system (LI-COR Biosciences, Lincoln, NE, USA), and images were processed using an image studio program. All antibodies were used at the manufacturer-recommended dilutions.

#### Real-Time qRT-PCR

Frozen day-60 liver and gastrocnemius muscle samples (from dosed limbs) were used to extract RNA using TRIzol Reagent according to the manufacturer’s instructions (Thermo Fisher Scientific, #15596026). The RNA was then treated with the TURBO DNA-free Kit (Thermo Fisher Scientific, #AM1907) to remove DNA contamination. A high-capacity RNA-to-cDNA kit (Thermo Fisher Scientific, #4387406) was used for reverse transcription to obtain cDNAs. qPCR was run using custom-designed Fam-labeled primers and probes targeting the transgene-c-myc junction (Thermo Fisher Scientific, #4448484). GAPDH was used as an endogenous control utilizing a VIC primer-limited expression assay (Thermo Fisher Scientific, #4451933).

#### gDNA Extraction and Real-Time PCR

AAV genome copies were measured using qPCR. The tissues were harvested in a manner that prevented cross-contamination, snap frozen in liquid nitrogen, and stored at −80°C until gDNA was extracted. gDNA was isolated from the liver, right calf, left calf, right quadriceps, left quadriceps, right inguinal lymph node, left inguinal lymph node, cervical spinal cord, and lumbar spinal cord using a DNeasy Blood & Tissue kit (QIAGEN, Valencia, CA, USA) according to the manufacturer’s instructions. gDNA concentrations were determined using the NanoDrop system (Thermo Fisher Scientific, Waltham, MA, USA). AAV genome copies present in gDNA were quantified by real-time PCR using the QuantStudio 3 Real-Time PCR System (Thermo Fisher Scientific, Waltham, MA, USA) according to the manufacturer’s instructions, and results were analyzed using the QuantStudio Design & Analysis v1.4.1 software. Briefly, primers and probes were designed to the polyomavirus simian virus 40 polyadenylation sequence (SV40) poly(A) region of the AAV vector used. A standard curve was performed using plasmid DNA containing the same SV40pA target sequence. PCR reactions contained a total volume of 50 μL and were run at the following conditions: 50°C for 2 min, 95°C for 10 min, and 45 cycles of 95°C for 15 s and 60°C for 1 min. DNA samples were assayed in triplicate. In order to assess PCR inhibition, the third replicate was spiked with plasmid DNA at a ratio of 100 copies per microgram of gDNA. If this replicate was greater than 40 copies per microgram of gDNA, then the results were considered acceptable. If a sample contained 100 copies or more per microgram of gDNA, it was considered positive for vgs. If a sample contained less than 100 copies per microgram of gDNA, it was considered negative for vgs. Vector copy numbers reported are standardized per microgram of gDNA. Assay controls include: a no template control (NTC) with acceptability criteria of <15 copies and an established study-specific standard curve slope range (±3 SD from three individual standard preparations and runs).

#### IFNγ-ELISpot Response to AAV1 and AAV8 Capsids

PBMCs were isolated before dosing and at day 60 post-injection and stimulated *in vitro* in R10 media supplemented with human interleukin (IL)-2 and IL-7 (1 ng/mL) and a complete set of AAV1 or AAV8 peptides (0.5 μg/mL) for 3 days. Then, cells were washed and resuspended in R10 media supplemented with human IL-2 and IL-7 (1 ng/mL) for 3 additional days. On day 6, cells were washed and left to rest overnight in R10 media. On day 7, the IFNγ-ELISpot assay was performed according to the manufacturer’s recommendations (Monkey IFNγ ELISpot^BASIC^, MABTech). PBMCs were stimulated *in vitro* with overlapping peptides spanning the AAV1 or AAV8 capsid VP1 sequences and divided into 3 pools (15-mers overlapping by 10 aa). A negative control consisted of unstimulated cells (medium only), whereas CD3 and CD28 stimulation was used as a positive control for cytokine secretion.

#### Statistical Analysis

Serum AAT (Western Data) was analyzed using a non-parametric one-way ANOVA (Kruskal-Wallis) on days 14 and 21 with a Dunn’s multiple comparisons test. We could not analyze day-60 results due to missing samples in the AAV8 groups. Significance was set at p ⩽ 0.05. ELISpot data were analyzed using a DFR(2×) test.

## Author Contributions

A.M.G.: study design, vector delivery, experimental work, data analysis, and manuscript preparation. G.G.: study design, experimental work, data analysis, and manuscript preparation. Q.T.: experimental work, data analysis, and manuscript preparation. G.-J.Y.: experimental work. D.R.K.: experimental work and vector production. G.W.: study design, experimental work, and manuscript preparation. J.B.: study design, experimental work, and manuscript preparation. K.E.C.: experimental work. A.M.K.: manuscript preparation and data analysis. C.M.: study design and manuscript review. L.G.C.: study design consultation. J.D.C.: study design and manuscript review. T.R.F.: study design, experimental work, data analysis, and manuscript preparation.

## Supplemental Information

Document S1. Tables S1–S3 and Figures S1 and S2

Document S2. Article plus Supplemental Information
