# Supplementary material for: Bridging from Intramuscular to Limb Perfusion Delivery of rAAV: Optimization in a Non-human Primate Study
Source: Mol Ther Methods Clin Dev. 2019 Feb 2;13:233–42. doi: 10.1016/j.omtm.2019.01.013 (PMC6383191; doi:10.1016/j.omtm.2019.01.013)
Supplement: Document S1. Tables S1–S3 and Figures S1 and S2 [file mmc1.pdf]

OMTM, Volume *13*

Supplemental Information

Bridging from Intramuscular to Limb Perfusion Delivery of rAAV: Optimization in a Non-human Primate Study

Alisha M. Gruntman, Gwladys Gernoux, Qiushi Tang, Guo-Jie Ye, Dave R. Knop, Gensheng Wang, Janet Benson, Kristen E. Coleman, Allison M. Keeler, Christian Mueller, Louis G. Chicoine, Jeffrey D. Chulay, and Terence R. Flotte

	FEMORAL ARTERY DELIVERY				PERIPHERAL VENOUS DELIVERY			
**Study**	**Arruda, 2004**	**Rodino-Klapac, 2007**	**Rodino-Klapac, 2010**	**Chicoine, 2013, 2014**	**Su, 2005**	**Toromanoff, 2008, 2009**	**Arruda, 2010**	**Le Guiner, 2014**
**Species (weight)**	Canine 12-22.5kg	Cynomologus Macaque 4-5kg	Rhesus Macaque 4-8kg	Rhesus Macaque	Canine 5-11kg	Cynomologus Macaque 3-5kg	Canine 8.7-24kg	Canine 8.7-24kg
**Route**	Infuse, Dwell, Flush - ipsilateral	Infuse and Dwell - ipsilateral - gastroc only	Infuse and Dwell - ipsilateral	Infuse and Dwell - ipsilateral - gastroc only	Hydrodynamic (ATVRX)	Hydrodynamic	Hydrodynamic (ATVRX)	Hydrodynamic
**Circulated Vector**	No	No	No	No	No	No	No	No
**Vector Gene**	LacZ and FIX	CMV eGFP	microdystrophin	CMV eGFP or MCK.GALGT2	CMV lacZ	human LEA29Y, cmEpo	cFIX	U7snRNA-E6/E8
**Dose - Vector**	1.7x10e12 - 3x10e12 vg/kg	2x10e12vg/kg	2x10e12vg/kg	2x10e12vg/kg	1x12e14 gc	5x10e12vg/kg	3x10e12 vg/kg	1x10e13 - 5x10e13 vg/kg
**Serotype**	AAV2	AAV8	AAV8	AAVrh.74	rAAV1	rAAV 1 > rAAV8	AAV2 and AAV6	AAV8
**Volume - Vector**	2.5ml/kg PBS with 10mM histamine, followed by 10ml/kg PBS	2ml PBS- over 60 sec	2.5ml/kg (gastroc only, tourniqu tight)	2.5ml/kg (gastroc only, tourniqu tight)	500ml PBS at 300mmHg over 20min	50ml/kg LRS over 5 minutes	20ml/kg over 3 minutes at 300mmHg	12ml/kg at 300mmHg or 6-7ml/kg at 10 or 35 ml/min
**Volume - Pre flush**	2.5ml/kg PBS with 10mM histamine,	2ml saline (pre tourniquet) 0.5ml/kg	2.5ml/kg (gastroc only, tourniquet snug but not tight)	2.5ml/kg over 1 minute	No	No	No	No
**Volume - Post flush**	15ml/kg PBS (Cimetidine and Benedryl after)	2ml PBS then tourniquet released	2.5ml/kg with tourniquet still tight over 60 sec	2.5ml/kg with tourniquet still tight then released	No	No	No	No
**Tourniquet pressure/level**	Proximal thigh (no pressure reported)	phlebotomy tourniquet above incision	Proximal and distal to gastroc	Proximal and distal to gastroc	Groin - until femoral pulse gone	350mmHg	Groin - until femoral pulse gone	Above Elbow - 310mmHg
**Limb Exsanguination**	No	No	No	No	No	No	No	Yes
**Vessel Clamping**	Arterial and venous clamping	No	No	No	No	No	No	No
**Arterial Catheter**	No size reported	3 french	3 french	3 french	No	No	No	No
**Venous Catheter**	No	No	No	No	Saphenous 20 gauge	Saphenous 22 gauge	Saphenous 14-18 gauge	Cephalic 20 gauge
**Vector Dwell Time**	15-20 minutes	10 minutes	10 minutes	10 minutes	20 minutes?	15 minutes	15 minutes	15 minutes

Supplemental Table 1. A Summary of Methods Used in Limb Infusion Studies Delivering rAAV to Large Animal Models, modified from Gruntman et al, Human Gene Therapy, 2015 [21].

Vector Route	Liver (vector genome copies/ug of DNA)	Liver - Total vector genomes	Muscle (vector genome copies/ug of DNA)	Muscle (Volume of Distribution)	Muscle (nuclei within transduced volume)	Muscle - Total vector genomes	Vector Genome Ratio % (total muscle : total liver x 100)
AAV1 IM	376,475	6.0×10^{10} copies	1,599,935	9.6 ml	2.40×10^{7} nuclei	1.37×10^{8} copies	0.22%
AAV1-VLP	1,961,980	3.2×10^{11} copies	783,635	500 ml	1.25×10^{9} nuclei	3.50×10^{9} copies	1.09%
AAV8-VLP	6,534,063	1.1×10^{12} copies	239,328	500 ml	1.25×10^{9} nuclei	1.07×10^{9} copies	0.15%
AAV1-IAPD	1,192,300	1.9×10^{11} copies	22,530	600 ml	1.50×10^{9} nuclei	1.21×10^{8} copies	0.064%
AAV8-IAPD	2,878,100	4.6×10^{12} copies	16,885	600 ml	1.50×10^{9} nuclei	9.04×10^{7} copies	0.019%

Supplemental Table 2. Estimated total vector genomes delivered to the lower extremity and liver with each dosing route. In calculating the total vg delivered, the volume of muscle transduced was directly estimated from the volume of the limb perfused by the vessels cannulated, or in the case of IM, by the volume of injection.

Dose Group	Animal #	IFNγ secretion to AAV capsid	
		Prior to dosing	Day 60
IM – AAV1	RA1598	-	-
	RA1683	-	-
	RA0764	-	-
VLP – AAV1	RA1567	-	-
	RA0770	-	-
	RA0332	-	+
IAPD – AAV1	RA1562	-	-
	RA1709	-	-
VLP – AAV8	RA1676	-	-
	RA1703	-	-
	RA1764	-	-
IAPD – AAV8	RA1660	-	-
	RA1664	-	+

Supplemental Table 3. IFNγ immune response to AAV1/AAV8 capsids measured by ELISpot. - : no IFNγ secretion; + : IFNγ positive response

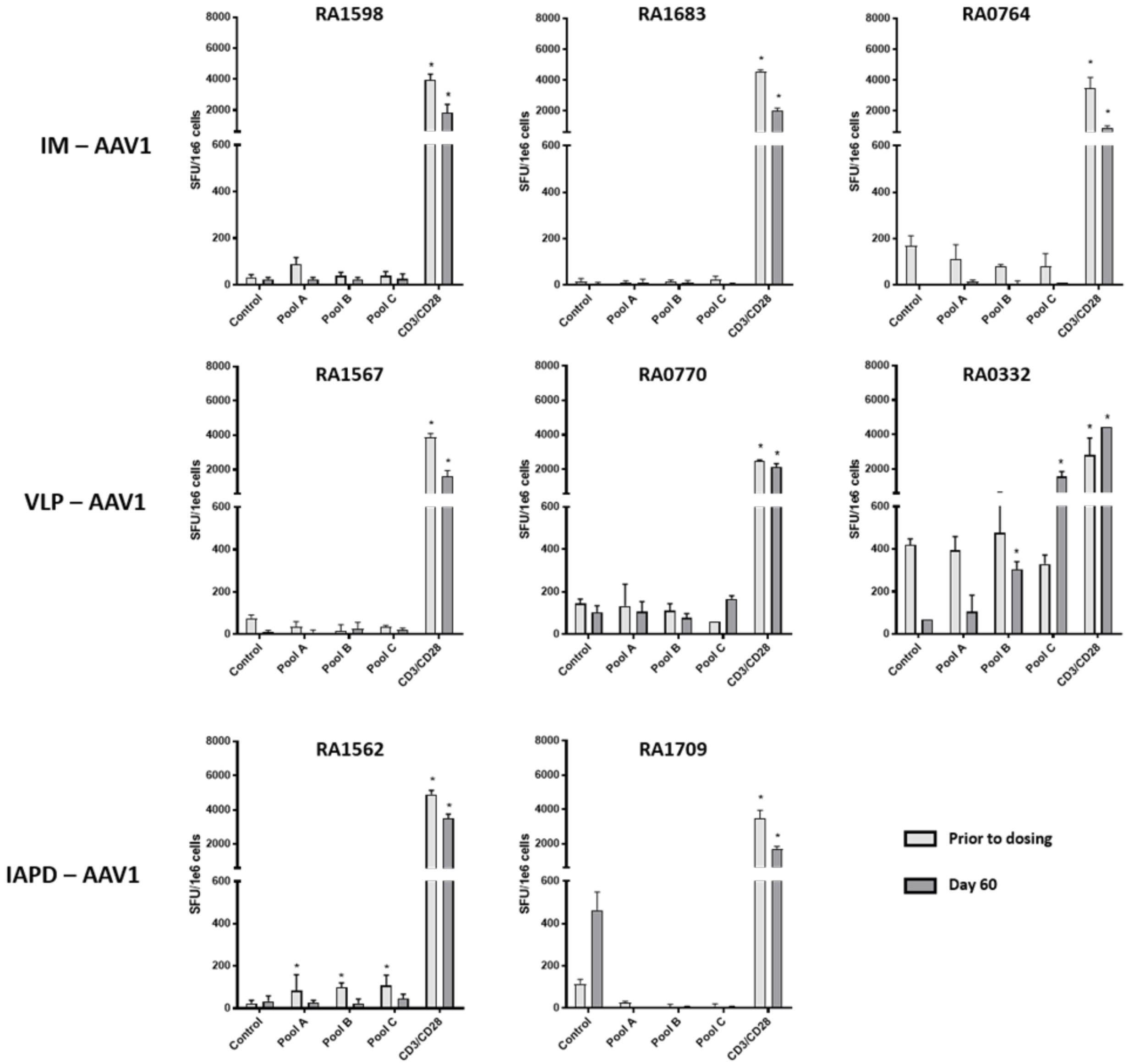

Supplemental Figure 1. IFNγ immune response to AAV1 capsid. Peripheral blood mononuclear cells collected prior to dosing and at necropsy (Day 60) were cultured 6 days before a 48-hour restimulation with AAV1 peptide pools. Comparing intramuscular (IM), intra-arterial push and dwell (IAPD) and venous limb perfusion (VLP) animals. Each graph represents a single animal. SFU: spot forming unit; * denotes a positive response (DFR(2X) test); CD3/CD28: positive control; Control: media only negative control.

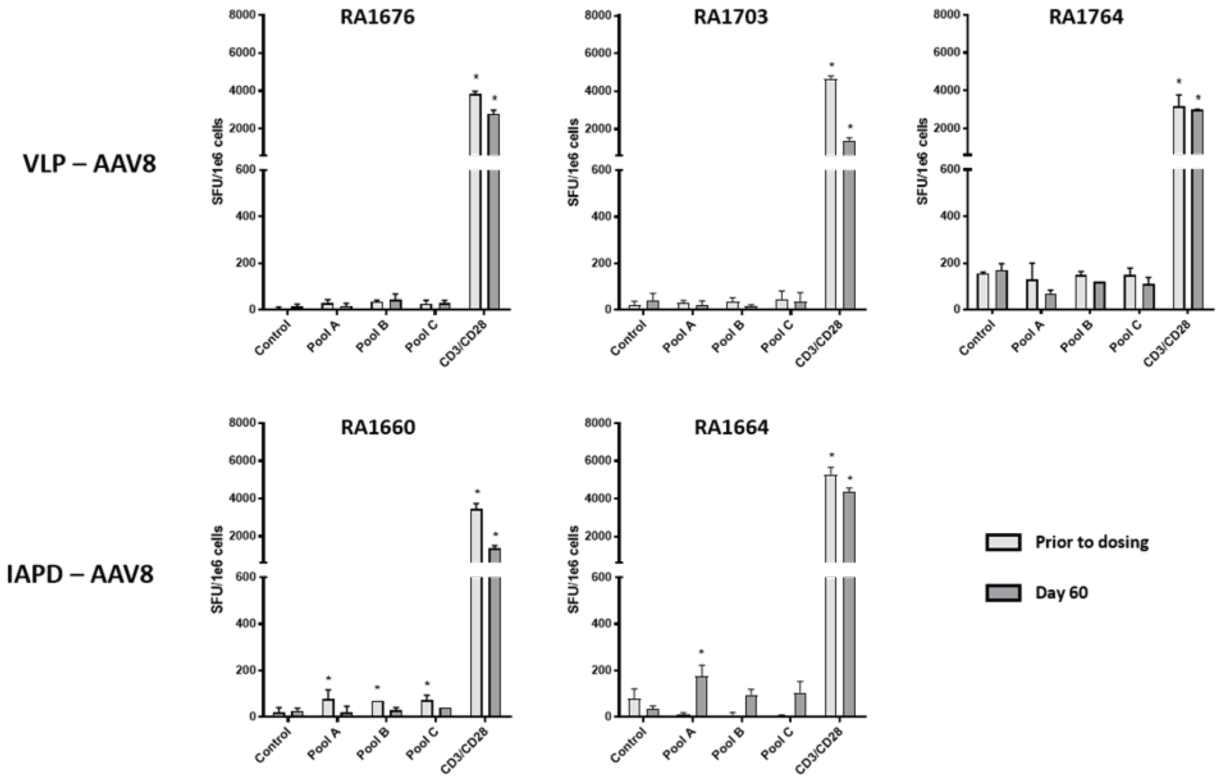

Supplemental Figure 2. IFNγ immune response to AAV8 capsid. Peripheral blood mononuclear cells collected prior to dosing and at necropsy (Day 60) were cultured 6 days before a 48-hour restimulation with AAV8 peptide pools. Comparing intramuscular (IM), intra-arterial push and dwell (IAPD) and venous limb perfusion (VLP) animals. Each graph represents a single animal. SFU: spot forming unit; * denotes a positive response (DFR(2X) test); CD3/CD28 : positive control; Control: media only negative control
